# Solution‐Processed Vertically Stacked Complementary Organic Circuits with Inkjet‐Printed Routing

**DOI:** 10.1002/advs.201500439

**Published:** 2016-02-19

**Authors:** Jimin Kwon, Sujeong Kyung, Sejung Yoon, Jae‐Joon Kim, Sungjune Jung

**Affiliations:** ^1^ Department of Creative IT EngineeringPohang University of Science and Technology (POSTECH)77 Cheongam‐RoNam‐Gu, PohangGyeongbuk790‐784South Korea

**Keywords:** 3D integration, inkjet‐printing, integrated circuits, organic field‐effect transistor, printed electronics

## Abstract

The fabrication and measurements of solution‐processed vertically stacked complementary organic field‐effect transistors (FETs) with a high static noise margin (SNM) are reported. In the device structure, a bottom‐gate p‐type organic FET (PFET) is vertically integrated on a top‐gate n‐type organic FET (NFET) with the gate shared in‐between. A new strategy has been proposed to maximize the SNM by matching the driving strengths of the PFET and the NFET by independently adjusting the dielectric capacitance of each type of transistor. Using ideally balanced inverters with the transistor‐on‐transistor structure, the first examples of universal logic gates by inkjet‐printed routing are demonstrated. It is believed that this work can be extended to large‐scale complementary integrated circuits with a high transistor density, simpler routing path, and high yield.

## 1 Introduction

Organic field‐effect transistors (OFETs) have been proposed for various applications, including microprocessors,^1^ programmable logic circuits,^2^ radio‐frequency identification circuits,^3^ and electronic skins.^4^ Most digital circuitry used in such circuits and systems requires no static power dissipation, wide noise margins, and high operational performance stability against transistor parameter variations. In silicon‐based microelectronics, these requirements were achieved by using logic circuits combining both p‐ and n‐type metal‐oxide transistors in a complementary symmetry configuration that drew almost zero power in standby mode.^5^ The same approach was adopted for the implementation of organic complementary circuits. Crone et al. fabricated clocked sequential logic circuits with small molecule complementary organic semiconductors such as hexadecafluoro‐copper‐phthalocyanine (F_16_CuPc) and *α*‐sexithiophene (*α*‐6T).^6^ Klauk et al. demonstrated ultralow‐power complementary circuits with F_16_CuPc and pentacene using plasma‐grown aluminum oxide and self‐assembled monolayer.^7^ All these logic circuits have been based on 2D planar structures with p‐ and n‐type transistors fabricated on the same floor.

Recently, a new structure was proposed in which two p‐type organic field‐effect transistors were vertically integrated^8^ and was then applied to the configuration of complementary transistors.^9^, ^10^, ^11^, ^12^, ^13^, ^14^ This approach is expected to increase the transistor density and minimize the interconnection paths among transistors in the implementation of complex functional digital or analog circuits. However, it must feature solution processability, low temperature, and nonaggressive solvents to facilitate processing on plastic and reduce costs.

In this study, we report the fabrication and measurements of solution‐processed vertically stacked complementary organic field‐effect transistors (VS‐COFETs) with a high noise margin. We demonstrate a new strategy to maximize the noise margin by matching the strengths of the p‐type organic FET (PFET) and n‐type organic FET (NFET) by independently adjusting the dielectric capacitance of each type of transistor. The ideally balanced VS‐COFETs were interconnected by inkjet printing to build logic circuits, such as inverters, NANDs, and NORs.

## 2 Results and Discussion

### 2.1 The Solution‐Processed VS‐COFET

**Figure 1:**
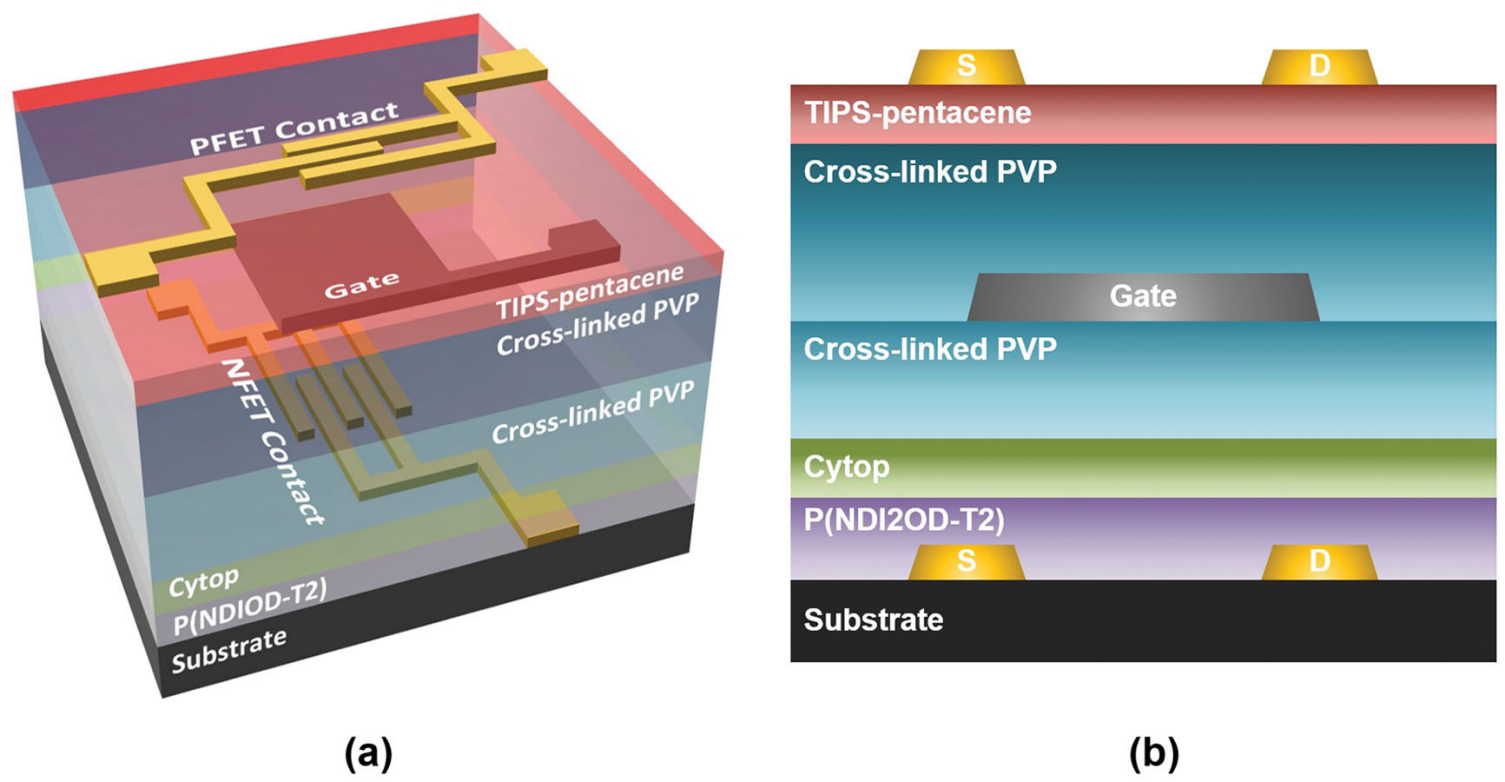
a) 3D and b) cross‐sectional schematic of the vertically stacked complementary field‐effect transistor (VS‐COFET).

The organic complementary logic circuits studied in this work were based on two vertically stacked OFETs. As shown in **Figure** **1**, a bottom‐gate top‐contact PFET is stacked on top of a top‐gate bottom‐contact NFET to form a complementary transistor. A gate electrode is shared by both transistors, which led to simpler fabrication without additional steps for another gate. This gate‐shared, transistor‐on‐transistor structure is ideally suited to implement universal logic circuits because NAND and NOR have two pairs of complementary transistors whose gate electrodes are connected.

The VS‐COFET consists of a total of eight layers including thermally evaporated metal electrodes, solution‐processed complementary organic semiconductors, and solution‐processed dielectric films. 6,13‐bis(triisopropylsilylethynyl) (TIPS‐pentacene) and poly{[*N*,*N*′‐bis(2‐octyldodecyl)‐naphthalene‐1,4,5,8‐bis(dicarboximide)‐2,6‐diyl]‐alt‐5,5′‐(2,2′‐bithiophene)} (P(NDI2OD‐T2)) were used as the PFET and NFET semiconductors, respectively. A selection of gate dielectric materials was found critical to vertically stacking the two transistors by the solution process. We deposited a thermally cross‐linked mixture of poly(4‐vinylphenol) and poly(melamine‐co‐formaldehyde) (PVP:PMF) for the PFET and a combination of amorphous fluoropolymer (Cytop) and PVP:PMF for the NFET. The combination of TIPS‐pentacene and PVP:PMF as well as P(NDI2OD‐T2) and Cytop showed good electrical performances in other studies.^15^, ^16^

**Figure 2:**
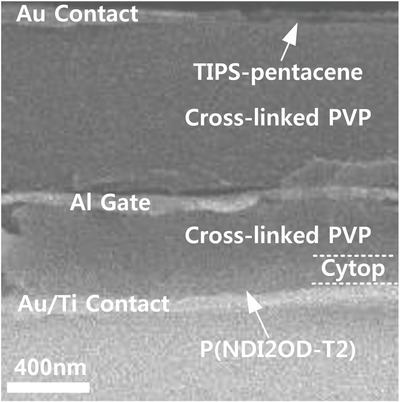
Cross‐sectional SEM image of the solution‐processed VS‐COFET.

**Figure 3:**
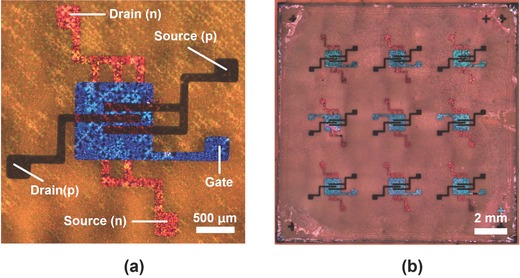
Top‐view images of a) the single VS‐COFET and b) nine VS‐COFETs fabricated on the glass substrate.

The thickness of the PVP:PMF dielectric layers in each FET was adjusted to balance the strength of the PFET and NFET in their complementary configuration. It is noteworthy that this fabrication process of the complementary transistors did not involve any high‐resolution patterning technique, which has been typically applied to separately define the p‐ and n‐type active semiconductor areas on the same surface.^17^, ^18^ **Figure** **2** shows a cross‐sectional scanning electron microscopy (SEM) image of the VS‐COFET. A top‐view polarized‐light microscopy image is shown in **Figure** **3**a. The crystalized TIPS‐pentacene film had approximately 100 μm‐size grains, whereas the P(NDI2OD‐T2) film had rather amorphous morphology (Figure S1, Supporting Information). The source and drain electrodes of the PFET on the top layer were placed perpendicular to those of the NFET on the bottom layer to minimize the possible interference between the two transistors. This electrode layout for a single device enabled us to develop universal logic circuits by post‐inkjet routing, which will be explained in the next section. The devices on the glass substrate are shown in Figure 3b.

### 2.2 Characteristics of the VS‐COFET Inverter

**Figure 4:**
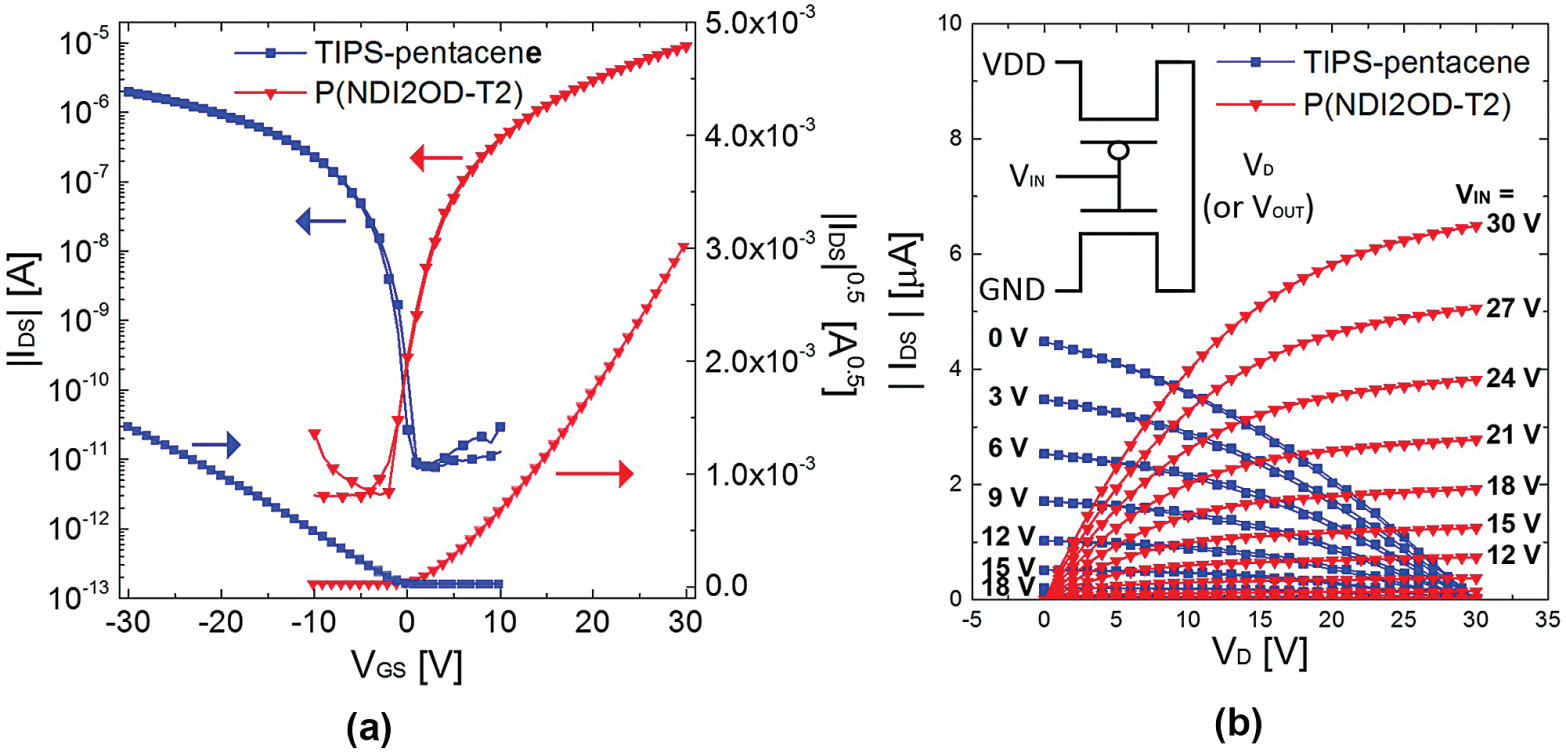
a) Complementary transfer curves of the VS‐COFET. b) Load line for the inverter operation of the complementary transistors. The symbol in the graph represents the inverter with the transistor‐on‐transistor structure.

The typical transfer characteristics of the vertically stacked PFET and NFET are shown in **Figure** **4**a. The PFET had channel width (*W*) of ≈970 μm and channel length (*L*) of ≈33 μm, whereas the *W* and *L* of the NFET were ≈1925 μm and ≈35 μm, respectively. To accurately extract the semiconductor parameters, we measured the channel geometries of all devices. The PFET had an average hole mobility (*μ*_p_) extracted in the saturation region of 0.048 cm^2^ V^−1^ s^−1^, an average threshold voltage (*V*_THp_) of −0.2 V, and a normal on/off current ratio of ≈10^4^. For the NFETs, the average saturation electron mobility (*μ*_n_) was 0.084 cm^2^ V^−1^ s^−1^, the average threshold voltage (*V*_THn_) was 7.2 V, and the normal on/off current ratio was ≈10^5^. The details for all the devices that we have tested are summarized in Figure S2 and Table S1 (Supporting Information). Figure 4b shows the output curves of the complementary transistor pair. It is often called the load line and it helps to establish the operating point of two nonlinear devices connected in series. By sweeping the drain node voltage (*V*_D_) of the complementary transistor from 0 to 30 V, the static channel currents (|*I*_DS_|) through the PFET and NFET were measured for the given input voltage (*V*_IN_) range of 0–30 V at 3 V steps.

**Figure 5:**
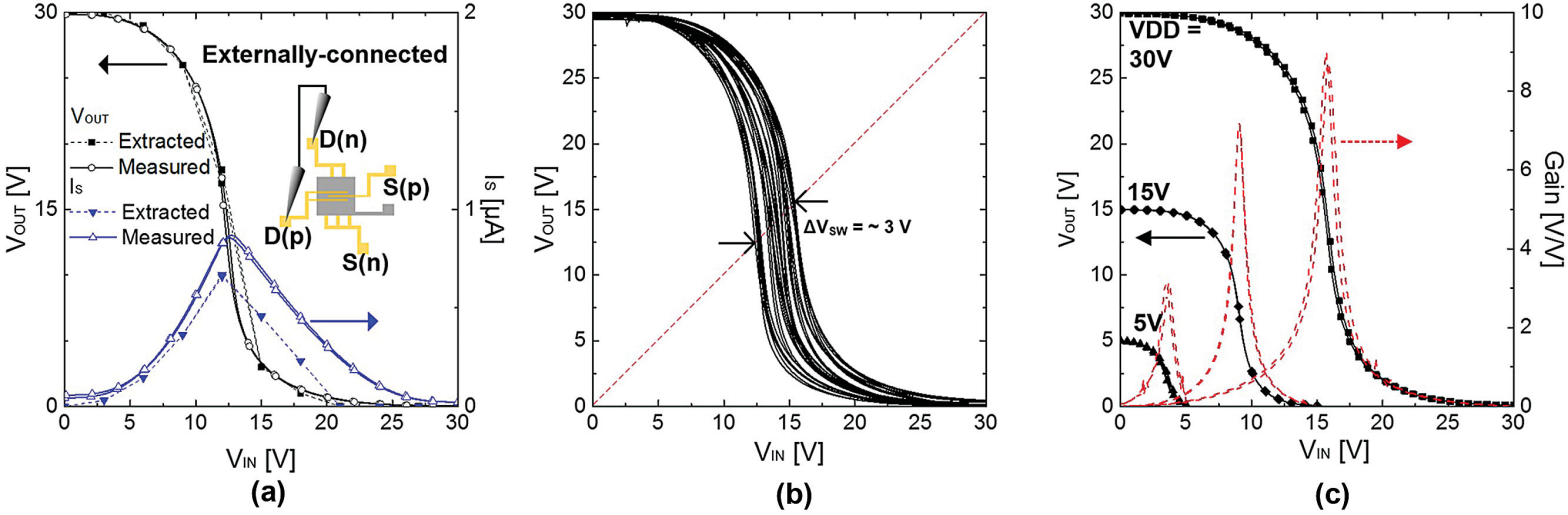
a) Comparison of the inverter operation extracted from the load line using experimental data. b) Output curves of 18 inverters with a switching threshold voltage of 14.5 ± 1.5 V. b) Inverter operation and gain under supply voltage of 5, 15, and 30 V.

The inverter curve was extracted from the load line and was then compared with the measured output curve, as seen in **Figure** **5**a. For the inverter measurements, the drain nodes of the NFET and PFET were externally connected using a probe station, in which the resistance of the connection path was negligible. The measured inverter curve showed almost identical operation with the inverter curve extracted from the load line graph, which implies that the individual transistors operated normally even when they were working together in the complementary configuration. To assess the reliability of the solution‐processed VS‐COFETs, we fabricated and tested 18 inverters (36 transistors). Figure 5b shows the output curves of the VS‐COFET inverters whose drain nodes were externally connected using the probe station. The inverters showed an average gain of 10.7 V/V and a maximum gain of 14.04 V/V. The switching threshold voltage (*V*_OUT_ = *V*_IN_) of the inverter curves was remarkably close to the ideal value of half of the supply voltage (VDD) (VDD/2 ≈ 14.5 ± 1.5 V). The VS‐COFET inverter, as seen in Figure 5c, operated over a wide range of supply voltage (5–30 V). The voltage gain improved from 3 to 9 V/V as the supply voltage increased from 5 to 30 V. It is noteworthy that the inverter showed clear operation at 5 V even though the VS‐COFET had relatively thick dielectric layers for such low voltage operation. This is attributed to the low turn‐on voltage (*V*_ON_) near 0 V for both PFET and NFET.

The switching threshold voltage defined as the point where *V*_IN_ =*V*_OUT_ is an important parameter that characterizes the input–output behavior of the complementary inverter circuit because it determines the static noise margin in digital circuits. Conventionally, the size of a transistor (typically the channel width) is adjusted for matching the difference in the performances of a PFET and a NFET.^19^, ^20^, ^21^ By choosing the appropriate ratio of the channel widths of the PFET and NFET, one can shift the transition region of the voltage transfer curve and set the switching threshold close to the ideal VDD/2 for maximizing the noise margin. However, changing the switching threshold by a considerable amount is not easy with the limited size of the transistors even when there is a largely unbalanced driving strength between two transistors, which can be often observed in organic electronic devices.

**Figure 6:**
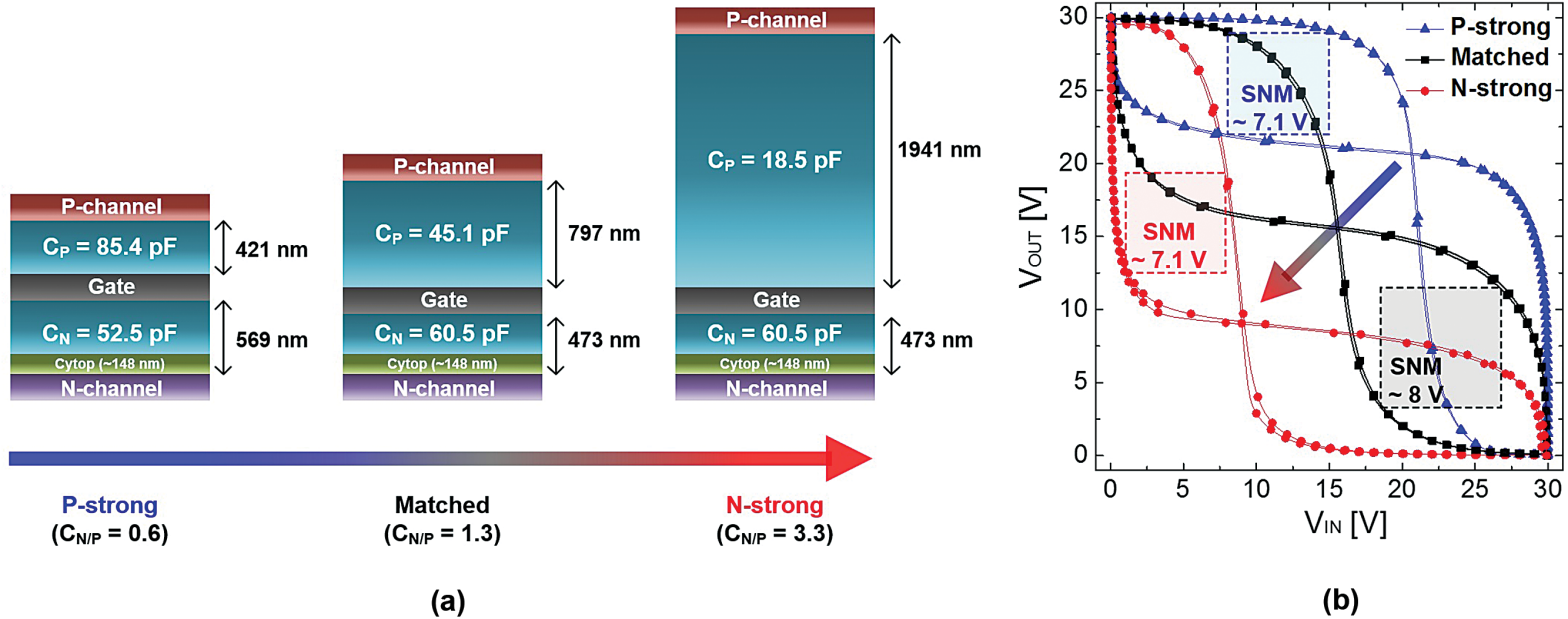
a) Three examples of VS‐COFETs with different dielectric capacitance ratios between the PFET and the NFET (*C*_N/P_ = *C*_N_/*C*_P_). b) Butterfly curves of the corresponding inverters with different driving strength.

Another way to match the strength of a PFET and an NFET is to independently adjust the dielectric capacitance of each transistor. However, this strategy cannot be adopted in conventional complementary circuits because a single gate dielectric should be shared by all the transistors on the same layer. The solution‐processed transistor‐on‐transistor structure studied in this work has, however, made it possible to use different kinds of polymeric dielectric materials with variable thickness for each transistors. It was observed that the threshold voltage, rather than carrier mobility, dominantly affected the switching point in the inverter curve with the PFET having lower |*V*_TH_| than the NFET (Figure 4a). With *W*/*L* of 1:2 for the PFET and NFET, the capacitance was accurately adjusted to carefully match the stacked complementary transistors. **Figure** **6**a shows examples of VS‐COFETs with three different dielectric capacitance ratios between the PFET and the NFET (*C*_N/P_ = *C*_N_/*C*_P_), achieved by varying the thickness of the PVP:PMF dielectric. The corresponding voltage transfer characteristics of three complementary inverters are shown with the calculated noise margins in Figure 6b. It can clearly be seen that the transition region of the inverter curve where the PFET and NFET are always saturated shifts with varying capacitance ratio. For the case of a stronger NFET over a PFET with a high capacitance ratio (*C*_N/P_ = 3.3), the transition region moved towards the ground voltage (GND) (red line), whereas the low capacitance ratio (*C*_N/P_ = 0.6) resulted in a stronger PFET pushing the switching threshold towards VDD (blue line). The driving strengths of the two transistors were balanced with *C*_N/P_ of 1.3 by setting the switching threshold at ideal VDD/2 (black line). The symmetrical input–output characteristics obtained from the controlled dielectric capacitance led to maximizing the noise margin to as high as 8.0 V. These results show precisely adjustable inverter chracteristics with high reliability, compared to previous studies^7^, ^9^, ^11^ (Table S2, Supporting Information).

### 2.3 VS‐COFET Logic Circuits with Inkjet‐Printed Routing

**Figure 7:**
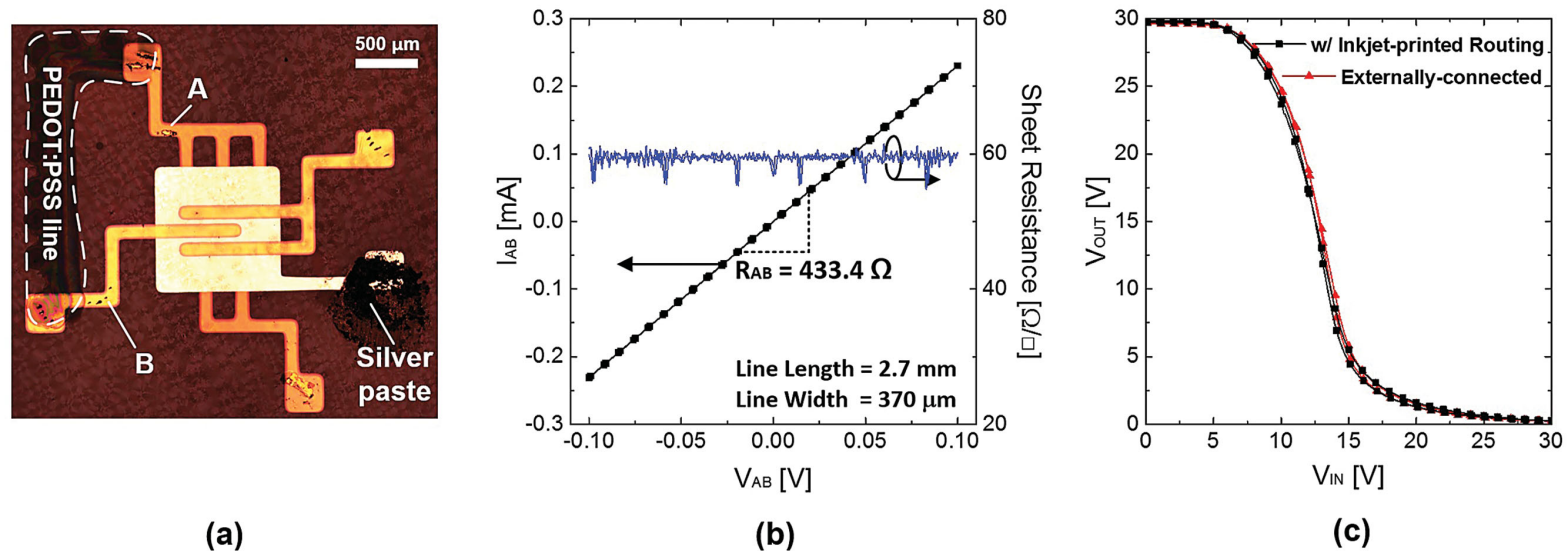
a) The VS‐COFET inverter with the inkjet‐printed routing (the dotted area). b) The *I*–*V* curve and sheet resistance of the inkjet‐printed wire. c) Comparison between an externally connected inverter and an inkjet‐routed inverter. The effect of the resistance of the inkjet‐printed wire is negligible.

Using symmetrically matched PFETs and NFETs, we fabricated logic circuits with inkjet‐printed routing. Highly conductive poly‐(3,4‐ethylenedioxythiophene):poly(styrenesulfonic acid) (PEDOT:PSS) with additives was printed for this purpose. The VS‐COFET inverter was implemented with the drain electrodes interconnected by a printed wire, as seen in **Figure** **7**a. The width of the PEDOT:PSS line was ≈370 μm and the length was ≈2.7 mm. The resistance between the A and B nodes was 433.4 Ω, as extracted from the *I*–*V* curve slope of Figure 7b, and the sheet resistance was approximately 60 Ω ◻^−1^. A negligible voltage drop (<500 μV) was observed during the operation of the inverter owing to the relatively low static current of the inverter (<1 μA). As shown in Figure 7c, the inkjet‐routed inverter showed almost identical DC operation with the externally connected inverter whose drain nodes were shorted by a probe station.

**Figure 8:**
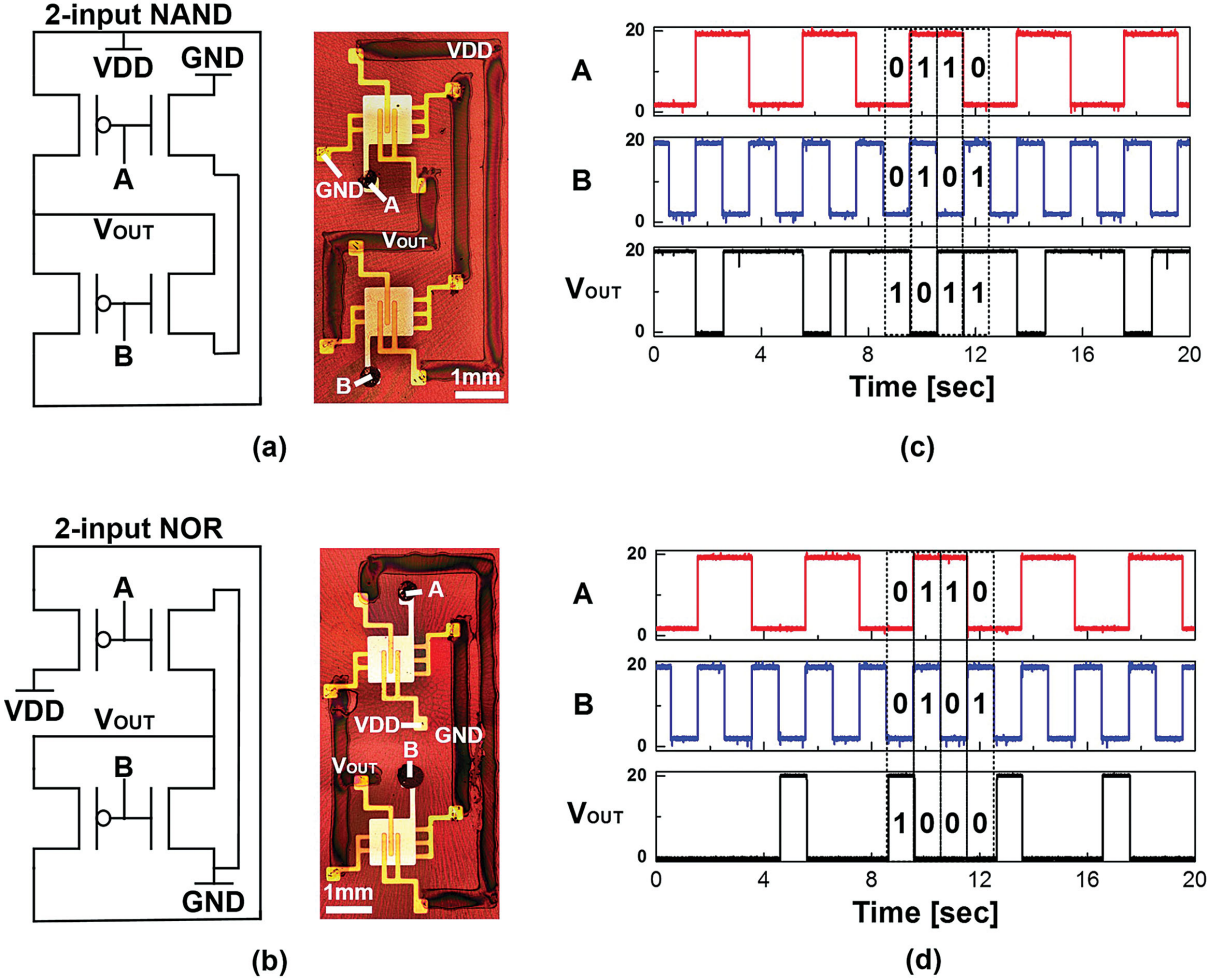
a,b) Circuit schematics and images of the 2‐input NAND and 2‐input NOR interconnected with the conductive wires. c,d) Dynamic measurement results of the NAND and NOR circuits.

Finally, the configuration of the universal logic gates was obtained by the same routing technique. Two pairs of VS‐COFETs (four transistors) were used to build each universal logic circuit. **Figure** **8**a,b shows the circuit schematics and images of a 2‐input NAND, and a 2‐input NOR interconnected with conductive wires. The circuit schematics show the device structure and inkjet routing lines. The dynamic measurement results of the logic circuits are shown in Figure 8c,d. Both NAND and NOR gates based on VS‐COFETs with inkjet‐printed wires showed correct logic operations.

## 3 Conclusion

In this work, we demonstrated solution‐processed vertically stacked complementary circuits with inkjet‐printed routing. The bottom‐gate PFET was fabricated on top of the top‐gate NFET and a gate electrode was shared in‐between. The VS‐COFET inverters showed reliable output curves with 10.7 V/V average voltage gain and had wide operation voltage (5 to 30 V). The structural advantage of the VS‐COFET allowed us to achieve symmetrical input–output characteristics by setting the switching threshold at the ideal VDD/2 by changing the dielectric capacitance ratio between the PFET and the NFET in their complementary configuration. We demonstrated the first examples of universal logic gates by interconnecting the vertically stacked transistors with the inkjet‐printed routing technique. We believe that by extending this work to large scales, complementary integrated circuits can be obtained with a high transistor density, simpler routing path, and high yield.

## 4 Experimental Section

*Device Fabrication and Measurement*: A glass substrate (Eagle XG) was sonicated in ultrapure water, acetone, and isopropanol for 10 min each. Au/Ti (3 and 40 nm) electrodes were thermally evaporated on the substrate for the n‐type OFET. P(NDI2OD‐T2) (purchased from Polyera) dissolved in 1,2‐dichlorobenzene (0.5 wt%) was spin‐coated at 3000 rpm for 60 s and annealed at 120 °C for 30 min. Cytop (1:1 in solvent) was spin‐coated at 3000 rpm for 60 s and annealed at 100 °C for 30 min. To modify the hydrophobic surface of Cytop, it was treated in Ar gas for 5 s with a DC plasma treater. The thickness of the Cytop film decreased 30 nm during the treatment. Then, PVP:PMF in PGMEA (12 wt%, 1:1 ratio) was spin‐coated at 2000 rpm for 60 s and was annealed at 150 °C for 30 min. The fabrication of the top‐gate NFET was completed by thermally depositing an Al gate electrode of 50 nm. On the top of the NFET, the PVP:PMF in PGMEA (20 wt%, 1:1 ratio) was spin‐coated at 2000 rpm for 60 s and annealed at 150 °C for 30 min for the dielectric layer of the p‐type transistor. 1 wt% TIPS‐pentacene (purchased from Ossila) in 1,2‐dichlorobenzene was spin‐coated at 2000 rpm for 60 s and annealed at 60 °C for 30 min. Au of 40 nm was thermally evaporated on the top contact electrodes.

The output and transfer characteristics of the VS‐COFET were measured by a probe system with a semiconductor parameter analyzer (Agilent 4156C). For measuring the NANDs and NORs, the output nodes were connected to a commercialized voltage buffer IC (CD4050BE, Texas Instruments) to provide enough current for full voltage swing. The amplitude of the voltage swing was halved without the buffer IC at the output stage. The operation voltage was limited by the maximum supply voltage (20 V) of the buffer. All the measurements were conducted under ambient environment.

*Printing Condition*: Highly conductive polymer PEDOT:PSS (Clevios PH 1000, Heraeus) was used for the printing. The solid content of the PH 1000 solution was 1–1.3% and had a PEDOT to PSS ratio of 1:2.5 by weight. 5 wt% of DMSO and 0.1 wt% of fluorosurfactant (Zonyl FS‐300, Sigma‐Aldrich) were added into the PEDOT:PSS to enhance conductivity and to lower the surface tension. The formulated ink was deposited to form a conductive line onto the substrate using a high‐resolution inkjet‐printing system (Jetlab II, MicroFab) with a piezoelectric printhead (MJ‐A, 40 μm nozzle, MicroFab). The ink was filtered with a 0.45 μm membrane filter before jetting. The speed of the ejected drops with diameter of ≈40 μm was ≈5 ms^−1^ and the jetting frequency was 500 Hz. The temperature of the printing substrate was maintained at 40 °C.

## Supporting information

As a service to our authors and readers, this journal provides supporting information supplied by the authors. Such materials are peer reviewed and may be re‐organized for online delivery, but are not copy‐edited or typeset. Technical support issues arising from supporting information (other than missing files) should be addressed to the authors.

Supplementary
