# Supplementary material for: Solution‐Processed Vertically Stacked Complementary Organic Circuits with Inkjet‐Printed Routing
Source: Adv Sci (Weinh). 2016 Feb 19;3(5):1500439. doi: 10.1002/advs.201500439 (PMC5067658; doi:10.1002/advs.201500439)
Supplement: Supplementary file 1 — Supplementary [file ADVS-3-0d-s001.pdf]

Copyright WILEY-VCH Verlag GmbH & Co. KGaA, 69469 Weinheim, Germany, 2016.

ADVANCED SCIENCE
Open Access

Supporting Information

for *Adv. Sci.*, DOI: 10.1002/advs. 201500439

Solution-Processed Vertically Stacked Complementary Organic Circuits with Inkjet-Printed Routing

Jimin Kwon, Sujeong Kyung, Sejung Yoon, Jae-Joon Kim, and Sungjune Jung**

Supporting Information

Solution-Processed Vertically Stacked Complementary Organic Circuits with Inkjet-Printed Routing

Jimin Kwon, Sujeong Kyung, Sejung Yoon, Jae-Joon Kim, and Sungjune Jung**

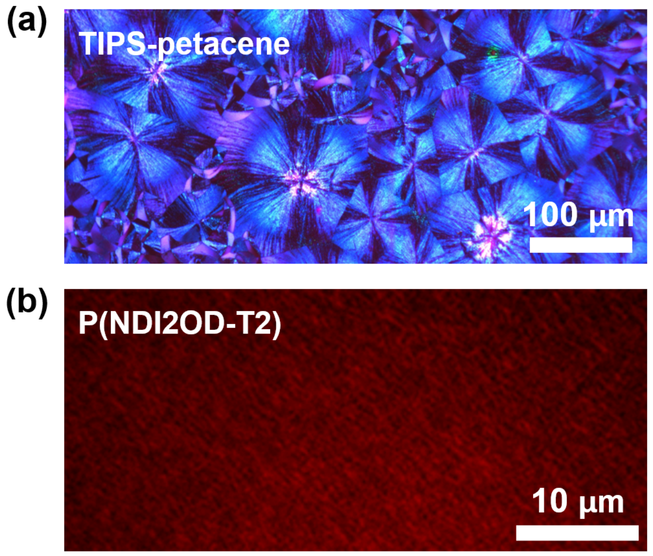

Figure S1. Polarized-light microscopy images of a) TIPS-pentacene film on cross-linked PVP layer and b) P(NDI2OD-T2) film on glass substrate.

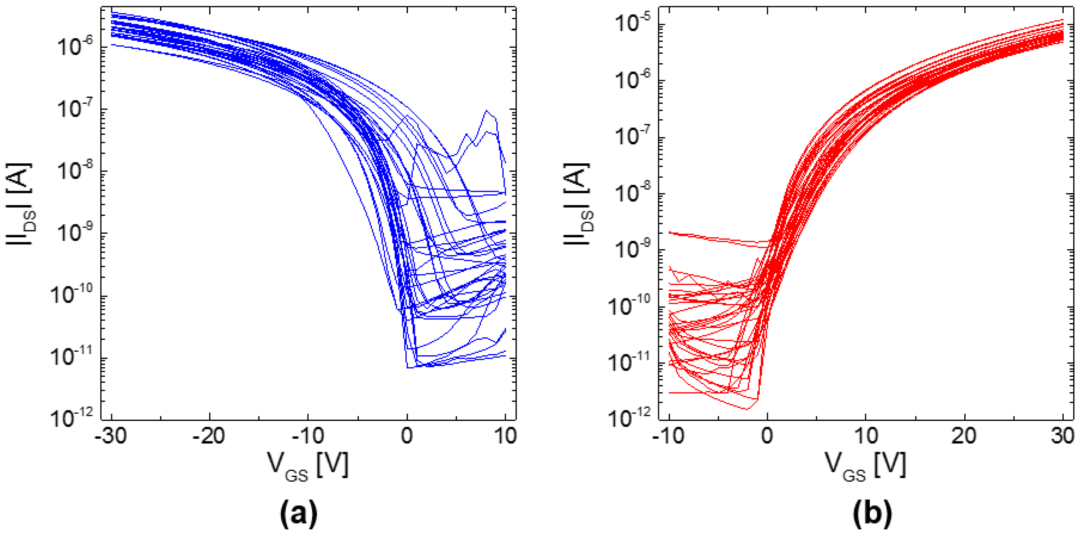

Figure S2. Transfer curves of a) PFETs and b) NFETs in VS-COFETs.

Table S1. Channel geometry (W_p/L_p and W_n/L_n), mobility (μ_p and μ_n), threshold voltage (V_{THp} and V_{THn}), and current on/off ratio ($|I_{ON}/I_{OFF|}$) of 18 VS-COFETs

Transistor #	PFET					NFET				
	W_p [μm]	L_p [μm]	μ_p [$cm^2 \cdot V \cdot s$]	V_{THp} [V]	$\log_{10}(\|I_{on}/I_{off}\|)$	W_n [μm]	L_n [μm]	μ_n [$cm^2 \cdot V \cdot s$]	V_{THn} [V]	$\log_{10}(\|I_{on}/I_{off}\|)$
1	934.8	34.6	0.0339	0.21	4.28	1939.2	35.9	0.0924	7.85	5.82
2	944.8	32.8	0.0330	1.12	3.50	1895.2	35.0	0.0805	9.13	5.50
3	948.2	39.3	0.0464	1.76	3.68	1892.0	39.8	0.0630	6.82	3.63
4	938.0	31.2	0.0554	−3.90	4.68	1969.2	33.6	0.0651	8.58	5.76
5	941.4	33.3	0.0251	−0.98	4.32	1915.2	35.6	0.0833	8.44	6.24
6	950.6	30.8	0.0453	−2.61	4.56	1955.6	34.6	0.0906	7.27	5.85
7	969.0	31.7	0.0689	−0.95	3.68	1938.8	33.4	0.1070	6.62	6.53
8	993.4	27.9	0.0406	3.87	3.23	1922.0	38.6	0.1004	8.08	5.23
9	970.0	31.7	0.0550	−1.43	2.31	1915.2	35.6	0.0607	6.57	5.36
10	980.0	32.4	0.0397	−2.37	4.09	1915.2	38.4	0.0834	8.00	4.67
11	978.2	32.4	0.0307	−0.06	3.87	1928.8	37.2	0.0882	7.81	6.65
12	1011.0	31.7	0.0374	6.50	3.67	1935.2	33.9	0.0709	6.19	4.85
13	985.8	30.5	0.0666	−1.28	5.68	1938.8	32.9	0.1145	5.71	5.05
14	980.8	33.1	0.0409	−0.95	5.42	1892.0	34.1	0.1004	6.89	6.46
15	972.4	32.6	0.0778	−0.88	3.00	1928.8	34.3	0.0597	4.74	6.45
16	995.8	32.6	0.0504	0.05	4.64	1932.0	31.1	0.0618	7.94	5.80
17	979.2	31.3	0.0508	−0.87	5.27	1912.0	37.4	0.0690	6.01	6.06
18	971.6	36.3	0.0574	−1.3	4.58	1922.0	37.3	0.1149	6.38	4.69
Average	969.2	32.6	0.048	−0.23	4.14	1924.8	35.5	0.084	7.17	5.59

Table S2. Comparison between complementary organic inverters.

Ref.	[7]	[9]	[11]	This work
**Structure**	Planar	Vertical	Vertical	Vertical
**Complementary / unipolar**	Complementary	Unipolar	Complementary	Complementary
**Dielectric process**	O_2 Plasma / SAM	Spin-coating	ALD	Spin-coating
**Semiconductor process**	Thermal Evap.	Thermal Evap.	Spin-coating.	Spin-coating
**Saturation μ [$cm^2 \cdot V^{-1} \cdot s^{-1}$]**	0.6 (p)<br>0.02 (n)	0.1 (p)<br>0.18 (p)	0.25 (p)<br>0.004 (n)	0.048 (p)<br>0.084 (n)
**VDD [V]**	3	20	6-8	5-30
**Max. gain [V/V]**	~100	13.4	26	14
**SNM**	~70 %	33 %	36 %	53 %
**V_{SW}/(VDD/2)[a)]**	0.73	~1.1	~1	~1
**Variation in V_{SW} [V]**	-	-	-	± 5%

a) V_S /(VDD/2) = 1 at the ideal switching point.
